# Supplementary material for: The Role of Plasma Cells as a Marker of Chronic Endometritis: A Systematic Review and Meta-Analysis
Source: Biomedicines. 2023 Jun 15;11(6):1714. doi: 10.3390/biomedicines11061714 (PMC10296269; doi:10.3390/biomedicines11061714)
Supplement: Supplementary file 1 [file biomedicines-11-01714-s001.zip › biomedicines-2296995-supplementary.pdf]

Supplementary Table S1

STUDY	PATIENT SELECTION	INDEX TEST	REFERENCE STANDARD	FLOW AND TIMING
Johnston-MacAnanny 2009	low risk	low risk	low risk	low risk
Zolghadri 2010	low risk	low risk	unclear risk	unclear risk
McQueen 2015	low risk	low risk	low risk	low risk
Chen 2016	low risk	low risk	unclear risk	low risk
Kitaya 2017	low risk	low risk	low risk	low risk
Song 2017	low risk	low risk	unclear risk	unclear risk
Zhang 2019	low risk	low risk	low risk	low risk
Hirata 2021	low risk	low risk	low risk	low risk
Herlihy 2022	low risk	low risk	low risk	low risk
